# Can survival analyses detect hunting pressure in a highly connected species? Lessons from straw-coloured fruit bats

**DOI:** 10.1016/j.biocon.2016.06.003

**Published:** 2016-08

**Authors:** David T.S. Hayman, Alison J. Peel

**Affiliations:** a Molecular Epidemiology and Public Health Laboratory, Hopkirk Research Institute, Massey University, Private Bag, 11 222, Palmerston North 4442, New Zealand; b Department of Veterinary Medicine, University of Cambridge, Cambridge CB3 0ES, UK; c Institute of Zoology, Zoological Society of London, Regent's Park, London NW1 4RY, UK; d Environmental Futures Research Institute, Griffith University, Brisbane, Queensland 4111, Australia

**Keywords:** *Eidolon helvum*, Demography, Hunting, Survival analyses, Bayesian population analyses

## Abstract

Animal behaviour, social structure and population dynamics affect community structure, interspecific interactions, and a species' resilience to harvesting. Building on new life history information for the straw-coloured fruit bat (*Eidolon helvum*) from multiple localities across Africa, we used survival analyses based on tooth-cementum annuli data to test alternative hypotheses relating to hunting pressure, demography and population connectivity. The estimated annual survival probability across Africa was high (≥ 0.64), but was greatest in colonies with the highest proportion of males. This difference in sex survival, along with age and sex capture biases and out-of-phase breeding across the species' distribution, leads us to hypothesize that *E. helvum* has a complex social structure. We found no evidence for additive mortality in heavily hunted populations, with most colonies having high survival with constant risk of mortality despite different hunting pressure. Given *E. helvum*'s slow life history strategy, similar survival patterns and rate among colonies suggest that local movement and regional migration may compensate for local excess hunting, but these were also not clearly detected. Our study suggests that spatio-temporal data are necessary to appropriately assess the population dynamics and conservation status of this and other species with similar traits.

## Highlights

• Straw-coloured fruit bat hunting pressure varies across African colonies.
• Estimated annual survival probability in five colonies across Africa was high (≥ 0.64).
• Social structure affects survival estimates, with male biases increasing estimates.
• Migration and movement may compensate for high local hunting pressure.
• Spatio-temporal studies are vital to assess mobile species conservation status.

## 1 Introduction

“At Avakubi [Democratic Republic of the Congo], November 19, 1909, a flock of perhaps 100 had taken shelter for the day beneath the limb of a large tree, some 60 feet above the ground, where they were shaded by a mass of epiphytic ferns and orchids, and formed one great squirming mass. … After watching their amusing struggles for a while, we fired both barrels of a gun into their midst. We were standing almost directly beneath and for a few seconds it simply rained bats, dead or wounded. … Hundreds of them are then slain by the natives, who are fond of eating these bats”.

Allen J A, Lang H, Chapin J (1917) The American Museum Congo expedition collection of bats (referring to *Eidolon helvum*, the straw-coloured fruit bat).

Demographic processes shape population dynamics and therefore have broad implications, for example on infection dynamics and sustainable harvesting capacity (Keeling and Rohani, 2008, Sandercock et al., 2011). Harvesting itself is hypothesized to shape demographic processes through various mechanisms (Sandercock et al., 2011). Under additive mortality, harvesting mortality has no effect on natural birth or death rates, and is additive to natural mortality in a linear fashion. Under compensatory mortality, density-dependent compensatory mechanisms (such as increased birth rates, decreased natural mortality, or increased movement) are evoked, meaning that harvesting adds no additional mortality to natural mortality rate. It is hypothesized that there should come a threshold at which these compensatory processes can no longer compensate and harvesting losses become additive (Sandercock et al., 2011). Understanding the contribution of compensatory or additive mortality processes is crucial for wildlife management and conservation so that offtake limits can be set to ensure harvesting does not deplete a population. Recent research has shown that fruit bats are a group of mammals that are hunted for food, sport or medicine in greater numbers than previously thought (Epstein et al., 2009, Harrison et al., 2011, Kamins et al., 2011, Mickleburgh et al., 2009). Correspondingly, studies on the sustainability of fruit bat bushmeat hunting are in their infancy compared with other terrestrial species.

Underlying demographic processes are often poorly elucidated in bats, even in common species. The straw-coloured fruit bat, *Eidolon helvum*, is one of the most common and widely distributed African bats, but is also widely hunted in parts of Africa (Anti et al., 2015, Carvalho et al., 2015, Kamins et al., 2011, Mickleburgh et al., 2009, Niamien et al., 2015). Demographic processes that affect whether hunting mortality may be additive or compensatory and their relevance for *E. helvum* fruit bats are considered under five different, but non-mutually exclusive, circumstances.

First, those species with slow life histories (long-lived with low fecundity, or so called ‘K-selected’ species), such as bats, primates, larger ungulates, and long-lived birds, are expected to suffer from additive mortality because they do not have the capacity to compensate for the additional mortality through reproductive surplus (Hamel et al., 2006, Sedinger et al., 2007, Sedinger et al., 2010). Hayman et al. (2012) estimated high annual adult survival rates and low fecundity for *E. helvum*, supporting expectations for a slow-life-history species.

Second, small or declining populations have limited compensatory capacity and are predicted to suffer from additive mortality (Bartmann et al., 1992). Significant *E. helvum* population declines have occurred in some areas of its sub-Saharan range, possibly because of habitat loss and over-harvesting for food and medicine (Mickleburgh et al., 2010, Mickleburgh et al., 2009, Perpetra and Kityo, 2009, Sodeinde and Soewu, 1999). Smaller, fragmented *E. helvum* populations also exist on a small number of offshore islands, including Pemba, off the Tanzanian coast (Hayman and Hill, 1971) and Príncipe, São Tomé and Annobón in the Gulf of Guinea (Juste and Ibanez, 1994). While Bioko Island in the Gulf of Guinea is close enough for bats to mix freely with the continental population, bats on Príncipe, São Tomé and Annobón islands are isolated and genetically distinct from one another (Peel et al., 2013). These smaller island populations may be more likely to suffer additive mortality in response to harvesting.

Third, migration and mixing among spatially structured populations can be a compensatory mechanism through demographic rescue (Cooley et al., 2009, Kvasnes et al., 2010). Continental *E. helvum* bats are seasonally migratory (Fahr et al., 2015, Funmilayo, 1979, Hayman et al., 2012, Mutere et al., 1980, Richter and Cumming, 2006, Thomas, 1983), and are capable of travelling > 2500 km across international borders and up to 370 km in a single night (Richter and Cumming, 2008). In contrast, the isolated nature of the island populations (or other fragmented populations) might also make them prone to additive effects.

Fourth, harvesting during or immediately after periods of natural mortality is more likely to be additive than if conducted before such periods (Boyce et al., 1999, Kokko, 2001, Ratikainen et al., 2008). Hunting pressure is spatially heterogeneous across *E. helvum*'s range (Kamins et al., 2011, Mickleburgh et al., 2009, Peel et al., 2016b). *E. helvum*'s migratory behaviour includes a tendency to vacate and arrive in roosts *en masse* (Fahr et al., 2015, Hayman et al., 2012, Peel et al., 2016b, Richter and Cumming, 2006, Thomas, 1983), resulting in the shifting seasonal presence of an apparently abundant resource for hunters along these migration routes.

Last, individual susceptibility to harvesting may vary with the type and timing of harvesting; for example, specific hunting methods intentionally or unintentionally target specific age or sex groups (Boyce et al., 1999). A variety of hunting methods have been documented for *E. helvum*, including shooting, slingshotting, capture in nets and hitting individuals that have fallen to the ground (Kamins et al., 2011, Mickleburgh et al., 2009, Peel et al., 2016b), however, few comprehensive data exist on how roost structure varies with age and sex, and in different locations or different seasons.

Empirical investigations to directly estimate the effect of harvesting within a target species or population are complicated by these multifactorial responses. For example, comparison of population counts among populations with differing harvest rates has been used to detect whether harvesting is likely to be additive or compensatory (Bodmer et al., 1997). However, in the case of compensatory mortality, population counts alone cannot separate the contributions of potentially contributing density-dependent processes (e.g. altered birth rates, survival and movement). The necessity to disentangle the various processes that contribute to population size when examining the effects of harvest on survival can be avoided by using mark-recapture techniques and harvest experiments (Bartmann et al., 1992, Cooley et al., 2009, Duriez et al., 2005, Obbard and Howe, 2008, Sandercock et al., 2011, Schaub and Lebreton, 2004, Servanty et al., 2010). These methods allow the effect of hunting to be anticipated by estimated harvesting rates and comparing those to survival rates under different harvesting pressures.

Logistical problems can preclude the possibility of using capture-recapture studies in some species, including bats. Instead, life table analyses enable population age structure, growth rate and survivorship patterns to be estimated and can allow for maturation of young and senescent mortality (Fieberg and DelGiudice, 2011, Kraus et al., 2013, Siler, 1979, Stolen and Barlow, 2003), thereby providing insight into the demographic processes of species for which cohort studies are not feasible. The flexible “Siler” model allows us to fit a function that includes maturation and senescence with a constant hazard (exponential) survival pattern as a base. The demographic processes relating to mortality for long-lived species, in which we might expect such maturation and senescence processes, can therefore be inferred in the absence of capture-recapture data.

Our expectations for long-lived species such as bats is that harvesting mortality will be additive. However, the highly connected colonies of *E. helvum*, determined through telemetry (Hayman et al., 2012) and inferred through population genetics (Peel et al., 2013), suggest that migration could be a compensatory mechanism that will overwhelm the local hunting pressure. In a companion paper, we provide new information on *E. helvum* colony sizes, hunting pressure, and age and sex structure for multiple colonies across tropical Africa (Peel et al., in Press). Here, we test hypotheses relating to hunting pressure and demography in this long-lived, yet highly mobile, species. First, we hypothesize that different host demographic structures and survival rates exist among regions and test whether this can be associated with different levels of local hunting pressure, or is likely related to other seasonal demographic and migratory processes. Second, we determine if mortality rates differ at different population sizes to make inferences regarding whether mortality through hunting is additive to natural mortality, or alternatively, that local movement and regional migration act as a compensatory mechanism. To test these hypotheses, we estimate harvest pressure and mortality risk across age classes from five different colonies across Africa and its outlying islands.

## 2 Methods

**Fig. 1:**
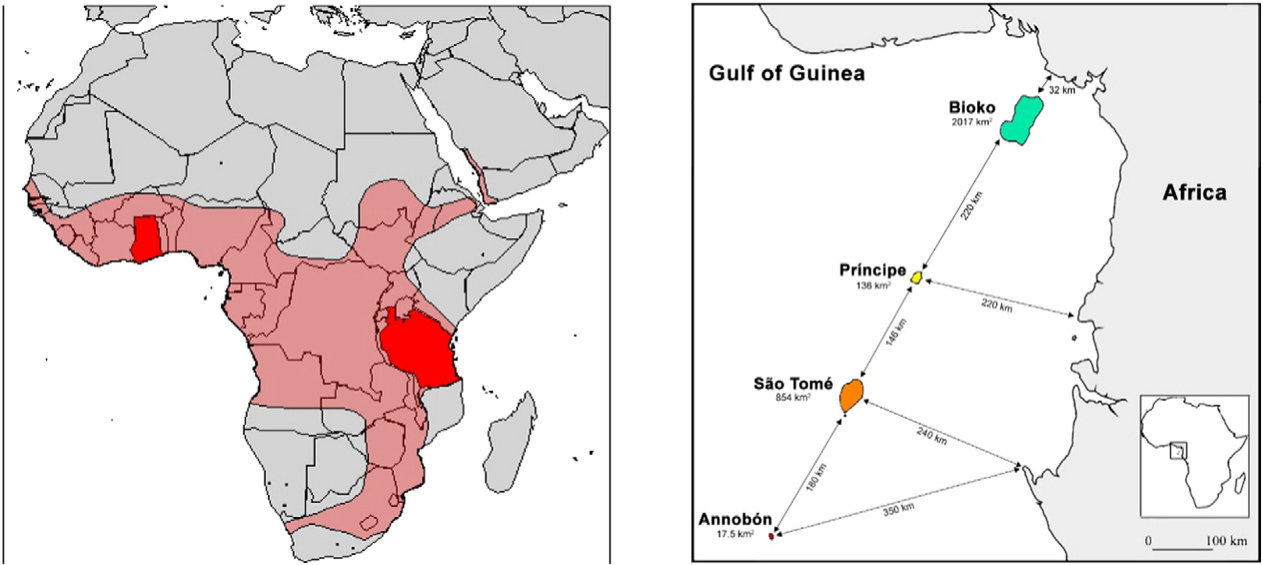
The distribution of the straw-coloured fruit bat, *Eidolon helvum*, red shading, with the continental sampling locations shown (red, left map). The Gulf of Guinea island sampling locations with the distances to the mainland (right map).

We used the background life history and hunting information for a subset of colonies of *E. helvum* across tropical Africa (Peel et al., 2016, and deposited in the online data repository, Dryad: doi:10.5061/dryad.2fp34). In particular, we present data from Accra (Ghana), Dar es Salaam (Tanzania), Morogoro (Tanzania), São Tomé (São Tomé and Príncipe), Príncipe (São Tomé and Príncipe), and Bioko (Equatorial Guinea) (Fig. 1). All fieldwork was undertaken under permits granted by national and local authorities (listed in the Acknowledgements section) and under ethics approval from the Zoological Society of London Ethics Committee (WLE/0489 and WLE/0467), using field protocols which followed ASM guidelines (Sikes et al., 2011).

Bats were either captured at the roost with mist nets as they departed the roost site at dusk (Ghana, Tanzania, Equatorial Guinea) or returned at dawn (Príncipe), or were shot by local hunters from roost or feeding sites (São Tomé). Morphometric (forearm length and weight) and demographic (age class, sex and reproductive status) data were recorded at the time of capture, and teeth were collected to determine age in years from tooth cementum annuli. Counting the cementum and dentine annuli was done by microscopy following histological preparation of tooth roots by Matson's laboratory, Milltown, Montana (Matson, 1993). Annuli were assumed to be deposited annually, based on other studies and the species strong seasonal migratory behaviour (Divljan et al., 2006, Hayman et al., 2012). Canine teeth were removed from dead bats killed for other purposes, including by hunters for meat, and processed as described elsewhere (Bodkin et al., 1997, Hayman et al., 2012, Matson, 1993, Peel et al., 2016b). Each age estimation (*n* = 233 bats) was scored with a certainty code: A: highest certainty of reported age (51% of samples), B: histological evidence supported a given age result ± 0.5–1.5 years (46% of samples), or C: tooth or section quality was too compromised to accurately age (3% of samples). The latter were not used in this study. The specific age structure of each roost was standardized according to the proportion of individuals caught within each age class. For example, in Bioko 84/105 (80%) of bats caught were less than 2 months old, but these were not aged through tooth cementum analyses. The proportion of 0-year-old bats was then corrected for in the tooth cementum data accounting for this capture bias.

In each location, roost or emergence counts were conducted following established techniques to estimate population sizes. Several methods were used, according to what was most appropriate in each location. The methods were daytime roost counts in Tanzania, Bioko, Ghana (Baranga and Kiregyera, 1982, Fahr et al., 2015, Hayman et al., 2012, Perpetra and Kityo, 2009) and emergence counts in Príncipe and São Tomé (Dallimer et al., 2006). These methods and results are described in detail elsewhere (Peel et al., in press).

Data on bat-human interactions were gathered via informal conversations and via questionnaire-based surveys (Table 1 and Appendix) (Peel et al., in press). Hunting pressure was then qualitatively categorised for each colony. Hunting was deemed ‘low’ in Dar es Salaam, Morogoro and Príncipe. Accra was estimated as ‘medium’ based on limited hunting in the immediate area, but high levels of hunting in nearby connected colonies (Kamins et al., 2011). São Tomé was subjectively deemed ‘high’ based on questionnaire responses, anecdotal reports from hunters and local residents and bat roosting behaviour, such as roosting away from human habitation (Peel et al., in press).

To estimate annual survival probability from age frequencies, and to test for variation in survival probability with age, we fit life table models to tooth age frequency data as performed elsewhere for mammal populations (Fieberg and DelGiudice, 2011, Kraus et al., 2013, Siler, 1979, Stolen and Barlow, 2003). Population growth rates (*λ*) cannot be estimated using our approach and so we assumed *λ* was constant (1) and assumed a stationary age structure (Hayman et al., 2012), but tested the sensitivity to this assumption by re-fitting the models with ln(*λ*) = − 0.1 (10% decline). As with the Accra colony data (Hayman et al., 2012), we tested five candidate models (below) based on models proposed by Siler (1979). This modelling approach assumes a constant baseline mortality risk operating throughout life and considers two additional factors, maturation (decreasing risk in early life) and senescence (increasing risk in later life). Annual probability of survival of mature animals at age *x* under constant baseline risk is given by the exponential model:

$$l_{x,2}=\exp\left(-a_{2}x\right)$$

and maturation and senescence elements are defined respectively by Gompertz models:

$$\begin{array}{l} l_{x,1}=\exp\left(\left(-a_{1}/b_{1}\right)\left(1-\exp\left(-b_{1}x\right)\right)\right)\\ l_{x,3}=\exp\left(\left(-a_{3}/b_{3}\right)\left(1-\exp\left(-b_{3}x\right)\right)\right) \end{array}$$

where *a*_*i*_ is the initial hazard for each element, *b*_*i*_ is the rate at which the hazard decreases or increases with age during maturation or senescence respectively, and *x* denotes age in years. Subscripts 1–3 denote, respectively, maturing, constant and senescing elements. Overall survivorship is then given by the product of desired components, such that the five models tested were constant risk (*l*_*x*_ = *l*_*x*,2_), maturing risk (*l*_*x*_ = *l*_*x*,1_ *l*_*x*,2_), senescing risk (*l*_*x*_ = *l*_*x*,2_ *l*_*x*,3_), both maturing and senescing risks (*l*_*x*_ = *l*_*x*,1_ *l*_*x*,2_ *l*_*x*,3_, aka the ‘Siler’ model) or both maturing and senescing risks without the constant risk (*l*_*x*_ = *l*_*x*,1_ *l*_*x*,3_). The inclusion of the latter model is an extension of previous work (Hayman et al., 2012). The model parameter estimates will allow greater understanding of the risk of mortality to different aged bats. For example, if younger bats are most affected by hunting, the initial hazard *a*_1_ will be higher in colonies with high hunting pressure. If hunting increases juvenile survival due to compensatory mechanisms, 1/*b*_1_ may be higher in heavily hunted colonies. If hunting affects all mature bats equally, *a*_2_ will be increased in those with heavy hunting pressure, and if hunting decreases, the overall age *a*_3_/*b*_3_ will be reduced as the risk of senescence reduces.

In contrast to our previous work using maximum likelihood (Hayman et al., 2012), we chose to fit these five non-linear models within a Bayesian framework because sample sizes were small for some colonies. We used a normal error structure with ‘uninformative’ (flat) priors. We chose normal (*N*) or uniform (*U*) distribution priors for the model parameters. Thus our full five-parameter Siler model for the hazard rates *μ* was

$$\mu \left[i\right]=ae^{\left(-a_{2}\mathrm{Age}\left[i\right]\right)}e^{\left(\left(-a_{3}/b_{3}\right)\left(1-e^{\left(b_{3}\mathrm{Age}\left[i\right]\right)}\right)\right)}e^{\left(\left(-a_{1}/b_{1}\right)\left(1-e^{\left(-b_{1}\mathrm{Age}\left[i\right]\right)}\right)\right)}$$

where

$$\begin{array}{l} P\left(a,a_{1},a_{2},a_{3},b_{1},b_{3}|y_{i}\right)\propto \prod_{i=1}^{n}N\left(y_{i}|\mu_{i},\frac{1}{\sigma^{2}}\right)\\ \;\;U\left(a|0,m_{a}\right)\\ \begin{array}{ccc} & & N\left(a_{1}|0,\sigma_{a_{1}}\right) \end{array}\\ \begin{array}{ccc} & & N\left(a_{2}|0,\sigma_{a_{2}}\right) \end{array}\\ \begin{array}{ccc} & & N\left(a_{3}|0,\sigma_{a_{3}}\right) \end{array}\\ \begin{array}{ccc} & & N\left(b_{1}|0,\sigma_{b_{1}}\right) \end{array}\\ \begin{array}{ccc} & & N\left(b_{3}|0,\sigma_{b_{3}}\right) \end{array}\\ \begin{array}{ccc} & & U\left(\sigma |0,m_{\sigma }\right) \end{array} \end{array}$$

Prior distribution dispersion parameter subscripts refer to the parameter of interest. We typically used *σ* = 100 for the uninformative normal prior distributions. We wrote all the code in R (R Development Core Team, 2013) and used the R2OpenBUGS package (Sturtz et al., 2005) to interface with OpenBUGS (Spiegelhalter et al., 2007). We ran three chains, for 10,000 iterations, discarding the first 1000 (10%) as burnin and did not thin the values at all, though we compared values and distributions with (every 10) and without thinning. We used the *nls2* function in the R package ‘nls2: Non-linear regression with brute force’ (Grothendieck, 2013) to help select initial conditions from a grid of parameter values, but chose randomly from values using appropriate distributions once appropriate initial conditions were found (not shown).

We estimated age-specific survival (*S*_*x*_) simply from the fitted risk models such that *S*_*x*_ = *l*(*x* + 1) / *l*(*x*). Results (see below) generally supported the assumption of constant survival with bat age (model 1, *l*_*x*_ = *l*_*x*,2_). Mean life expectancy calculated from annual survival probabilities (*S*) using the formula life expectancy = − 1 / ln(*S*) from the constant risk model *l*_*x*_ = *l*_*x*,2_. Colony-specific age-constant survival rates were therefore plotted against colony size estimates and hunting pressure estimates to assess whether any evidence existed for compensatory or additive mortality rates, and against sampling phase and colony sex ratio to assess the impact of seasonal dynamics and migration on survival estimates. The latter were further explored using linear mixed-effects models with age in years as the response variable, sex as a fixed-effect parameter and location and/or phase as a random effect, using the *lmer* function in the *lme4* package (Bates et al., 2013) in R. The age data (*y*) was transformed through a square-root transformation of the data and maximum likelihood used to fit the model to data, such that *y* ~ 1 + *Sex* + (1 |* Location*) and, separately, *y* ~ 1 + *Sex* + (1 |* Phase*). Further details regarding the data for these variables are in our companion paper (Peel et al., in press). For all results for the Bayesian Siler risk-based models 95% credible intervals are given, elsewhere 95% confidence intervals.

## 3 Results

Tooth age data were available from six colonies of straw-coloured fruit bats. We were able to fit the models to data from five colonies: Accra (Ghana); Dar es Salaam (Tanzania), Morogoro (Tanzania) São Tomé (São Tomé and Príncipe) and Príncipe (São Tomé and Príncipe). Data from Bioko (Equatorial Guinea) were highly biased towards very young bats (< 2 months) and we were unable to fit the models to these data (Peel et al., in press).

All Markov chain Monte Carlo (MCMC) chains converged well in the Bayesian analyses once appropriate initial conditions were chosen and the models ran. Example model outputs and all parameter estimates with credible intervals are shown in the Appendix.

**Fig. 2:**
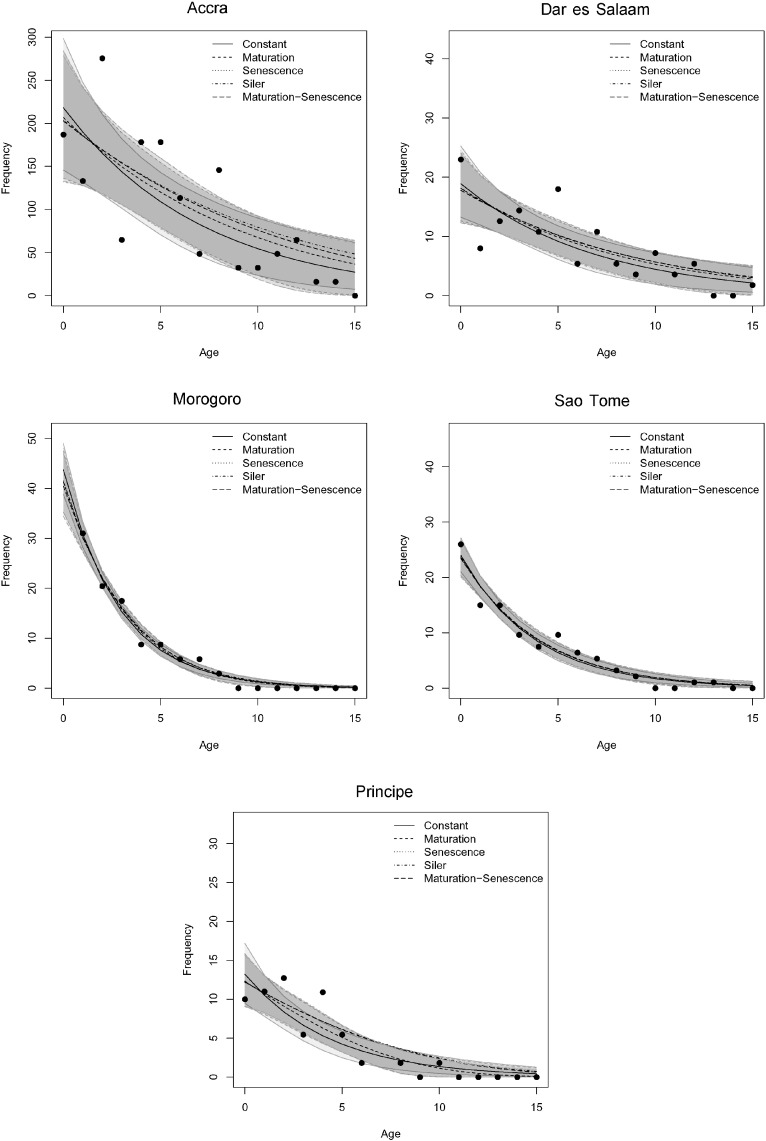
The hazard model fits to the standardized tooth cementum annuli data for each colony. Overall survivorship is constant risk (*l*_*x*_ = *l*_*x*,2_, solid line), maturing risk (*l*_*x*_ = *l*_*x*,1_*l*_*x*,2_, dashed line), senescing risk (*lx* = *l*_*x*,2_*l*_*x*,3_, dotted line), and constant, maturing and senescing risks (*lx* = *l*_*x*,1_*l*_*x*,2_*l*_*x*,3_, dot-dashed line) and maturing and senescing risks only (*lx* = *l*_*x*,1_*l*_*x*,3_, long-dashed line). 95% credible intervals are shown in grey.

The constant risk model had the strongest support (lowest DIC values) in all but the Principe population (Spiegelhalter et al., 2002) (Table 2). For Príncipe, the maturation/senescence model (*l*_*x*_ = *l*_*x*,1_ *l*_*x*,3_) was most strongly supported over all other models (Table 2, underlined). However, even when there was support for a better model over the constant model, the effect size was small, with overlapping credible intervals for the predicted age frequencies (Fig. 2). Fits of each model to the data are shown in Fig. 2. Comparison of parameter estimates also provides little support for differing parameters in the Siler model among locations with most maturation and senescence related parameter estimates including zero (Appendix). We also tested the assumption that these populations were at constant population size during the lifetimes of these bats by altering ln(*λ*) from 0 to − 0.1 (i.e. a negative population growth rate). The estimated parameters varied little by including this change (Appendix). Therefore, we used the constant survival rates for further analyses for simplicity and to allow comparison among sites.

**Fig. 3:**
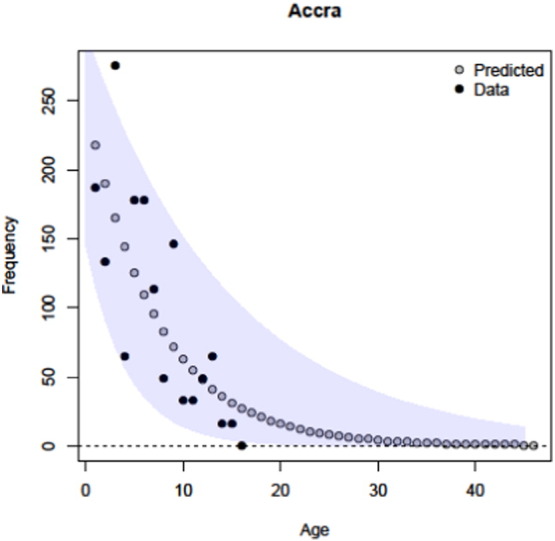
Predicted age frequencies for the Accra, Ghana colony using the constant survival rates estimated for *Eidolon helvum*. 95% confidence intervals are shown.

In all analyses our estimates of survival across age groups support that *E. helvum* is a relatively long-lived species, with a mean life expectancy estimates across the colonies ranging from 2.3 to 6.8 years, as can be seen after converting the model results to annual survival probabilities (Table 3). Using the best model results for the location with the highest survival, Accra in Ghana, and predicting the age distribution, our model predicts that individuals may live up to 30 years of age (Fig. 3), consistent with studies reporting individuals up to 21 years (DeFrees and Wilson, 1988).

**Fig. 4:**
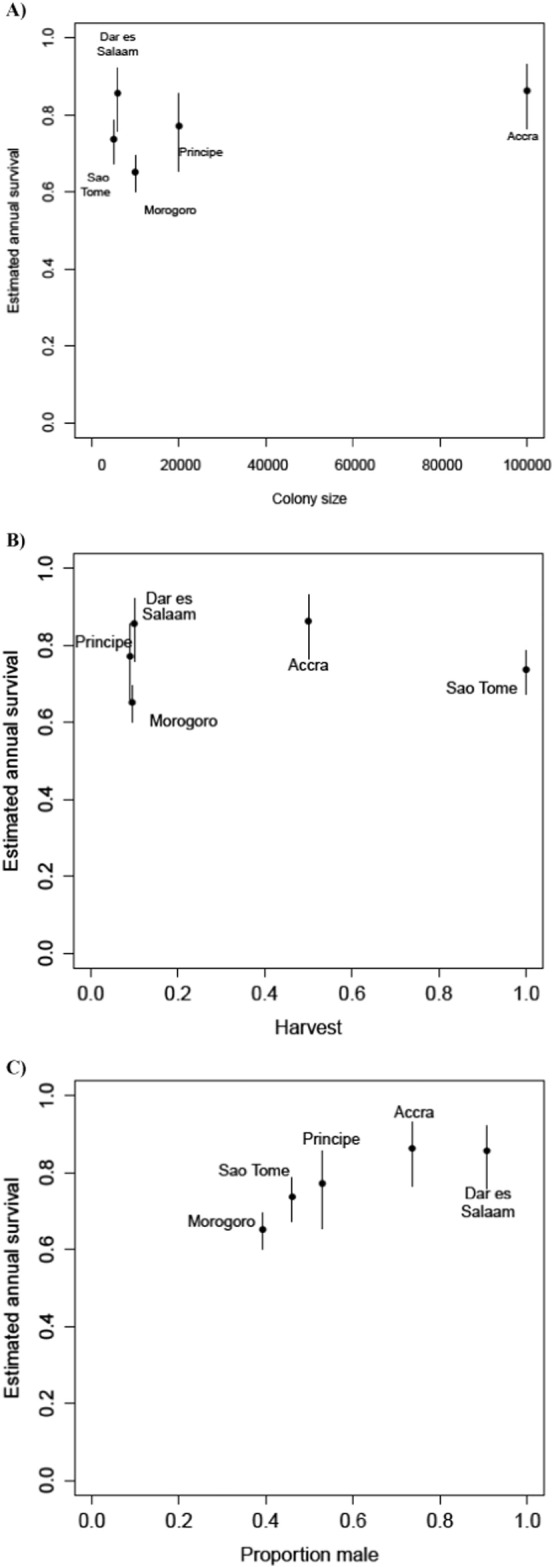
Constant survival rates estimated for *Eidolon helvum* from five different locations with differing colony size (A), harvest rates (B, ranked 0 to 1 subjectively on hunting pressure, Peel et al., in press), and proportion of males (C). Constant survival rate estimates and proportion male across five colonies were correlated (*F*-statistic 15.28, *p* < 0.05). Mean estimates with 95% credible intervals are shown for each colony.

Analyses determining if harvest mortality was additive or led to compensatory survival were inconclusive. Neither colony size nor hunting was statistically supported as an explanatory covariate for survival. Since compensatory and additive mortality represent density-dependent and density-independent natural mortality, respectively, if mortality is compensatory and in the absence of social facilitation effects, lower survival rates are expected with larger colony sizes (Fig. 4). In comparison, rates are not expected to vary with population size when mortality is additive (Burnham and Anderson, 1984). Here, plotting constant survival rates against population size showed no evidence for density-dependent (compensatory) mortality in *E. helvum* (Fig. 4). The São Tomé population size is likely to be underestimated (Peel et al., in press), but it is not known by how much and if that would strengthen support for additive mortality. In turn, if natural mortality rates cannot compensate for increased harvest, then survival is expected to decline with harvest intensity (Anderson and Burnham, 1976). Alternatively, if compensatory changes in natural mortality can occur, then constant survival rates will be observed in spite of increased harvest rates. Plotting constant survival rates against harvest rates suggested that *E. helvum* mortality rates could show compensation for hunting pressure. However, a caveat associated with this plot is that hunting estimates were only qualitatively estimated relative to population size.

A significant relationship was observed between colony sex ratios and survival rates (*F*-statistic 15.65, p 0.029; Fig. 4), however there is no relationship between sampling phase and survival rate (Appendix).

## 4 Discussion

Studies on the effects of hunting on bat populations are in their infancy. *E. helvum*, like other fruit bats with slow life histories, is poorly adapted to compensate for hunting demographically (Hayman et al., 2012, McIlwee and Martin, 2002). Previous harvest models of fruit bats have assumed additive hunting mortality (Epstein et al., 2009), yet this assumption has not been examined. Here, we explored the factors expected to determine *E. helvum*'s response to harvesting pressure.

Survival rates and associated life expectancies calculated here for *E. helvum* bats in Tanzania (Morogoro, 0.65, 0.60–0.69 95% CI, Dar Es Salaam 0.85, 0.76–0.92), São Tomé (0.74, 0.67–0.79 95% CI) and Príncipe (0.77, 0.65–0.86 95% CI) were comparable with previous estimates from Accra (0.83, 0.73–0.93 95% CI) (Hayman et al., 2012). The estimate for Accra (0.86, 0.77–0.93 95% CI) was slightly different to that previously estimated (0.83, 0.73–0.93 95% CI), due to stochastic Bayesian modelling and increased sample size, but were within reported confidence limits. Our mean life expectancy estimate across colonies is 4.5 years (range 2.3–6.7), but our models suggest individuals may live up to approximately 30 years old in the wild (Fig. 3).

Data on mechanisms governing natural mortality on fruit bats are limited, but McIlwee and Martin (2002) argued against density-dependence being a major contributor, partly due to reported causes of mortality for flying-foxes not being related to, or dependent on, the size or density of the populations. Our results showed no evidence for density-dependent mortality operating within colonies, irrespective of the presence of hunting (Fig. 4), suggesting the colonies studied here are not at carrying capacity. This is consistent with reports of population declines due to other threatening processes such as habitat loss (Mickleburgh et al., 2009), and thereby greater susceptibility to additive effects of hunting (Bartmann et al., 1992, Sandercock et al., 2011). Exploring our model's sensitivity to changes in population growth rate (*λ*) does little to help us understand these population dynamics and determine if the colonies are in decline (Fig. S3).

*E. helvum*'s mobility might help counteract additive effects of hunting-related mortality. The species moves among colonies within a region (Hayman et al., 2012) and regionally across international borders during migration (Richter and Cumming, 2008), resulting in a panmictic population structure across sub-Saharan Africa (Peel et al., 2013). This daily mobility and migratory capacity allows flexibility to utilize changing availability of food resources, maintenance of a wide and diverse gene pool, and avoidance of threats such as hunting. Individual animal movements within the panmictic continental population likely compensates for localised hunting-related mortality.

Conversely, smaller island populations with less migratory and rescue capabilities may be more likely to experience additive effects of hunting. Similar physical environments in the paired island system of São Tomé and Príncipe would be expected to result in little difference in natural survival between the two islands. However, differences in hunting pressure exist and while constant survival rates were similar, the Príncipe colony showed evidence for both maturation and senescence (Table 3). With an absence of migration and heavy hunting pressure, Príncipe may be the most natural system in our data set. Given this, it would suggest harvesting may remove maturation and senescence signals by altering demographic structure, leading to more constant risk across age classes. Alternatively, results could differ between the two islands due to sampling biases. The breeding seasons of *E. helvum* are out of phase on the islands, and sample collection was conducted via hunting at dispersed, small day-roosts soon after the beginning of the birth pulse in São Tomé versus via overnight mist-netting outside of breeding and mating seasons in a large colony in Príncipe (Peel et al., in press). Follow-up studies in these two island systems would be valuable in providing insight into what age structure of long-lived bats should look like, and the conservation implications of the heavy hunting currently being experienced in São Tomé. Studies of declining and smaller isolated populations may also be used to determine if social facilitation through Allee effects exist, thus leading to intermediate colony sizes having the highest survival through the positive Allee effects and lack of inhibitory mortality when at carrying capacity.

Heterogeneity among individuals in their susceptibility to harvest may affect the overall population response. Here, a greater proportion of males predicted greater survival probability. We speculate that rather than males being longer lived, this might reflect seasonal variation in social structures via non-random roosting and migration, resulting in unmeasured colony ‘types’ within sampled populations (Peel et al., in press). During periods where the majority of individuals in a colony undertake seasonal migration, the two colonies here with the highest proportions of males and survival rates (Accra and Dar es Salaam) maintain ‘resident’ male colonies where perhaps older, more dominant males are able to remain in the colony and maintain their roosting territory. In Ghana, females migrated prior to males and more males remained resident (Hayman et al., 2012) and male biases were observed in small roosts in São Tomé (Peel et al., in press), however sample sizes were insufficient to support separate analyses by colony type. Greater male survival has been reported in North American little brown bats, *Myotis lucifugus*, however other studies report greater female survival being greater, with non-random bat roosting noted as a confounding factor (Keen and Hitchcock, 1980). Other robust bat survival studies including large capture-recapture studies (e.g. Sendor and Simon, 2003, Hayman et al., 2012) have not found differences in survival between sexes.

Hunting methods such as netting and shotguns at roosts are presumed to be random (Epstein et al., 2009), though only if the targeted colony is an unbiased representation of the total population. With the heterogeneity in colony types observed here, the effect of even ‘random’ hunting methods is likely to vary according to the location and timing of hunting (Peel et al., in press). A particular region where hunting occurs may only ever see migrating populations with a female- and younger-age class bias. Alternatively, some hunting methods may be truly biased. Hunting using wire-mesh fruit traps during the mating season in São Tomé and Príncipe results in a male-biased capture if a female is caught first (Peel et al., in press).

Heterogeneity in survival and vulnerability to harvest can mimic compensation and mask detection of additive mortality (Sandercock et al., 2011). Heterogeneity in colony structure and susceptibility to hunting is an important factor to control for, and further characterisation of the relationship between age and sex biases and hunting pressures should be a focus for future studies. Relative seasonality of natural mortality and harvesting can change the way the harvesting mortality affects a species' population dynamics (Boyce et al., 1999). No data exist on seasonality in natural mortality in *E. helvum*. Migration is typically assumed to be costly, with cost positively associated with migration distance (Lok et al., 2015), so higher natural mortality of *E. helvum* peaks could be expected during and soon after migration. Overlap of hunting with migration might therefore result in additive mortality. Strong seasonality of bat hunting has been reported in other species (Brooke and Tschapka, 2002, Epstein et al., 2009, Struebig et al., 2007), coinciding with seasonal peaks in abundance due to migration. Kamins et al. (2011) reported strong hunting seasonality (November to March) in Ghana, coinciding with the beginning of the gestation period through to the beginning of the birthing period and northerly migration in the region (Hayman et al., 2012, Thomas, 1983), so peaks in hunting naturally coincide with migratory influxes.

A caveat in our analyses is we standardize the age count data of each colony to account for tooth analyses being conducted on a subset of individuals caught (Peel et al., in press). The standardisation may smooth over the subtler effects of maturation and senescence, though we feel this is unlikely to be a major effect as the Principe data still showed maturation and senescence (Table 2). An alternative explanation for the finding that there is constant risk and relatively high survival rates across locations may be because life table analyses can be biased if there is not a stable age distribution (Williams et al., 2002). Changes in hunting pressures and methods may affect inferences from life table analyses, depending on whether the populations are in decline, hunting is more or less random, or if it is variable in intensity. In rapidly declining populations, an analysis of age structure may fail to detect the decline, especially if harvesting affects all age classes. Our analyses were relatively insensitive to changes in population growth rate (*λ*, Fig. S3), highlighting the need for longitudinal studies.

Despite observations and analyses presented here involving considerable effort and contributing to sparse and patchy data published on this species, we were unable to test all our hypotheses. Sex was the strongest predictor of survival, though we propose this is likely to be a function of colony ‘type’ rather than inherently longer survival of males. The lack of knowledge of social structure, roost location and colony sizes of *E. helvum*, and other harvested bat species, limits the capability to determine the risk from over-hunting and warrants further studies across large spatial scales.

Based on all the contributing factors discussed, we hypothesize natural mortality in *E. helvum* includes maturation with reduced mortality risk and senescence, as observed in the least hunted colony from Príncipe. Our study failed to find substantial differences in survival rates among sites with different hunting and migration rates. However, we hypothesize that hunting is largely additive to natural mortality in *E. helvum* but that the large continental distribution with regional variation in hunting pressure and migration among source and sink colonies may allow some compensation. Local movement and regional migration and compensation they may provide highlights the conservation benefit for maintaining habitat continuity in migratory species.

Our inability to detect the impact of hunting through life table methods raises an important issue for conservationists, because *E. helvum* is extremely mobile, long-lived, and highly hunted. *E. helvum* was listed as Near Threatened in 2008 due to significant population declines (Mickleburgh et al., 2010). The potential implications for ecosystem functioning through pollination and seed dispersal resulting from even small declines of a common and widespread species like *E. helvum* are substantial (Gaston and Fuller, 2008). Our studies suggest that robust longitudinal, multi-site and indeed multi-national studies are required to determine if this species is being over-harvested. In the absence of such studies, *E. helvum* and other similar species may be driven into decline without us knowing it.

## Contributions

Conceived the research: AJP, DTSH

Analysed the data: AJP, DTSH

Wrote the manuscript: AJP, DTSH

## Figures and Tables

Distribution data were from the IUCN Red List (http://www.iucnredlist.org/) and maps from rworldmap (South, 2011).

**Table 1:** Hunting and population size details for colonies used in survival analyses, sorted by hunting pressure.

Location	Estimated hunting pressure	Number of bats caught	Proportion male	Estimated population size
São Tomé, São Tomé and Príncipe	High	102	0.91	9000^a^
Accra, Ghana	Medium	1518	0.39	100,000^b^
Dar es Salaam, Tanzania	Low	130	0.74	5000
Príncipe, São Tomé and Príncipe	Low	61	0.46	24,000
Morogoro, Tanzania	Low	101	0.53	10,000

a Likely substantially underestimated (Peel et al., in press).

b Range from 4000 to 1,000,000 (Hayman et al., 2012).

**Table 2:** Deviance information criterion (DIC) values for each model for each data set analysed. Support for models with greater than two DIC units are indicated by bold text. For Príncipe, the ‘Both’ model with maturation and senescence only was better than all other models (underlined). Lowest DIC values are in italics.

	Dar es Salaam	Morogoro	Accra	São Tomé	Príncipe
Constant	*93.88*	***55.53***	*176.90*	***66.10***	77.32
Maturation	94.81	57.93	177.59	68.46	79.10
Senescence	94.81	57.53	177.59	68.63	79.09
Maturation-senescence	94.88	58.15	178.11	69.13	*76.93*
Siler	95.29	58.12	178.18	68.53	78.06

**Table 3:** Mean life expectancy calculated from annual survival probabilities (*S*) using the formula life expectancy = − 1 / ln(*S*), where *S* was estimated from the constant risk model *l*_*x*_ = *l*_*x*,2_ (see Methods).

Location	Mean annual survival probability (S)	Mean life expectancy (years)
Dar es Salaam	0.86 (0.76–0.92)	6.71
Morogoro	0.65 (0.60–0.69)	2.32
Accra	0.86 (0.77–0.93)	6.71
São Tomé	0.74 (0.67–0.79)	3.26
Príncipe	0.77 (0.65–0.86)	3.87

## Appendix A Supplementary data

Supplementary material. Image 1
