# Supplementary material for: Can survival analyses detect hunting pressure in a highly connected species? Lessons from straw-coloured fruit bats
Source: Biol Conserv. 2016 Aug;200:131–9. doi: 10.1016/j.biocon.2016.06.003 (PMC4965785; doi:10.1016/j.biocon.2016.06.003)
Supplement: Supplementary file 1 — Supplementary material. [file mmc1.docx]

**Appendix**

**Table S1**

Mean parameter values (*a*, *a_1_*, *a_2_*, *a_3_*, *b_1_*, *b_3_*) for each model for each dataset estimated in a Bayesian framework (95% credible intervals are given). The model type ‘Mat_Sen’ is the combined maturation and senescence model.

| Model | Location | *a* | *a_1_* | *a_2_* | *a_3_* | *b_1_* | *b_3_* |
| --- | --- | --- | --- | --- | --- | --- | --- |
| Constant | Dar Es Salaam | 19  (13-25) | NA | 0.145  (0.077-0.241) | NA | NA | NA |
|  | Morogoro | 44  (39-49) | NA | 0.35  (0.305-0.4) | NA | NA | NA |
|  | Accra | 218  (146-299) | NA | 0.139  (0.068-0.235) | NA | NA | NA |
|  | São Tomé | 24  (21-27) | NA | 0.264  (0.215-0.326) | NA | NA | NA |
|  | Príncipe | 13  (9-17) | NA | 0.228  (0.144-0.346) | NA | NA | NA |
| Maturation | Dar Es Salaam | 18  (12-24) | 0.058  (-0.091-0.196) | 0.061  (-0.102-0.207) | NA | -0.009  (-0.181-0.187) | NA |
|  | Morogoro | 41  (35-47) | 0.152  (0.031-0.285) | 0.153  (0-0.306) | NA | -0.059  (-0.186-0.094) | NA |
|  | Accra | 207  (136-284) | 0.058  (-0.1-0.193) | 0.047  (-0.125-0.199) | NA | -0.022  (-0.194-0.182) | NA |
|  | São Tomé | 24  (20-27) | 0.113  (-0.016-0.254) | 0.138  (-0.01-0.272) | NA | -0.001  (-0.155-0.172) | NA |
|  | Príncipe | 12  (9-16) | 0.114  (-0.012-0.232) | 0.023  (-0.165-0.219) | NA | -0.117  (-0.295-0.108) | NA |
| Senescence | Dar Es Salaam | 18  (13-24) | NA | 0.061  (-0.103-0.204) | -0.059  (-0.198-0.088) | NA | 0.009  (-0.188-0.185) |
| Senescence | Morogoro | 41  (35-48) | NA | 0.153  (-0.002-0.308) | -0.153  (-0.288--0.031) | NA | 0.057  (-0.096-0.188) |
| Senescence | Accra | 207  (136-284) | NA | 0.048  (-0.127-0.201) | -0.057  (-0.193-0.096) | NA | 0.021  (-0.185-0.194) |
| Senescence | São Tomé | 24  (20-27) | NA | 0.138  (-0.011-0.273) | -0.113  (-0.254-0.017) | NA | 0.002  (-0.175-0.155) |
| Senescence | Príncipe | 12  (9-16) | NA | 0.022  (-0.165-0.215) | -0.114  (-0.23-0.016) | NA | 0.118  (-0.112-0.297) |
| Mat_Sen | Dar Es Salaam | 18  (12-24) | 0.053  (-0.105-0.193) | NA | -0.054  (-0.192-0.103) | -0.011  (-0.187-0.184) | 0.011  (-0.189-0.188) |
| Mat_Sen | Morogoro | 40  (34-47) | 0.145  (0-0.282) | NA | -0.146  (-0.285--0.003) | -0.035 (-0.182-0.153) | 0.032  (-0.159-0.181) |
| Mat_Sen | Accra | 203  (133-278) | 0.043  (-0.13-0.185) | NA | -0.047  (-0.185-0.124) | -0.017 (-0.194-0.188) | 0.02  (-0.183-0.196) |
| Mat_Sen | São Tomé | 23  (20-27) | 0.122  (-0.016-0.258) | NA | -0.123  (-0.26-0.014) | -0.005 (-0.165-0.182) | 0.003  (-0.186-0.161) |
| Mat_Sen | Príncipe | 12  (9-16) | 0.058  (-0.151-0.217) | NA | -0.059  (-0.217-0.152) | -0.065  (-0.28-0.176) | 0.066  (-0.174-0.281) |
| Siler | Dar Es Salaam | 18  (12-24) | 0.039  (-0.128-0.192) | 0.037  (-0.132-0.198) | -0.04  (-0.194-0.127) | 0.001  (-0.173-0.194) | -0.001  (-0.193-0.176) |
| Siler | Morogoro | 41  (35-48) | 0.103  (-0.054-0.256) | 0.106  (-0.057-0.273) | -0.101  (-0.256-0.059) | -0.025  (-0.187-0.168) | 0.023  (-0.175-0.182) |
| Siler | Accra | 204  (132-284) | 0.033  (-0.147-0.187) | 0.024  (-0.148-0.193) | -0.034  (-0.189-0.147) | -0.009  (-0.192-0.192) | 0.009  (-0.197-0.195) |
| Siler | São Tomé | 24  (20-27) | 0.081  (-0.069-0.234) | 0.095  (-0.067-0.252) | -0.081  (-0.238-0.068) | 0.008  (-0.163-0.196) | -0.007  (-0.198-0.165) |
| Siler | Príncipe | 12  (9-16) | 0.056  (-0.158-0.224) | 0.006  (-0.175-0.193) | -0.057  (-0.225-0.151) | -0.06  (-0.276-0.179) | 0.059  (-0.177-0.275) |

**Figures**

i)

ii)

**Fig. S1.** Density plots of the posterior probabilities (black line) informed by the data, with uninformative prior probabilities superimposed (red line), for each parameter in the survival model (i: Accra, Ghana, ii: São Tomé). Parameters: (*a*) initial hazard for each element, (*a_1_*) maturing element, (*a_2_*) constant element, (*a_3_*) senescing element, (*b_1_*) rate at which the hazard decreases with age during maturation, (*b_3_*) rate at which the hazard increases with age during senescence

**Fig. S2.** Relationship between constant survival rates and sampling phase (months since beginning of the birth pulse) across five colonies.

**Fig. S3.** The hazard model fits to the standardised tooth cementum annuli data for Accra. Overall survivorship in this example is constant risk (*l_x_* = *l_x,2_*, solid line) with and without a 10% decline (*ln(λ) = -0.1*). 95% credible intervals are shown in shaded colours.
